# A comprehensive review of natural products in rheumatoid arthritis: therapeutic potential and mechanisms

**DOI:** 10.3389/fimmu.2025.1501019

**Published:** 2025-05-08

**Authors:** Yuli Han, Shujing Chen, Changqing Liu, Huihui Sun, Zhaoyu Jia, Jiaxin Shi, Jin Li, Yanxu Chang

**Affiliations:** ^1^ State Key Laboratory of Component-based Chinese Medicine, Tianjin University of Traditional Chinese Medicine, Tianjin, China; ^2^ Haihe Laboratory of Modern Chinese Medicine, Tianjin University of Traditional Chinese Medicine, Tianjin, China

**Keywords:** rheumatoid arthritis, natural products, therapeutic potential, targets, mechanisms

## Abstract

Rheumatoid arthritis (RA) is a classic autoimmune disease caused by a combination of genetic and environmental factors. The multiple and comprehensive pathologies involving the whole body’s immune system and local organs and tissues make it challenging to control or cure them clinically. Fortunately, there are increasing reports that multiple non-toxic or low-toxicity natural products and their derivatives (NP&TDs) have positive therapeutic effects on RA. This review focuses on the potential mechanisms of NP&TDs against RA and aims to provide constructive information for developing rational clinical therapies. Active components of NP&TDs can play therapeutic and palliative roles in RA through multiple biological mechanisms. These mechanisms primarily involve immunosuppressive, anti-inflammatory, autophagic, and apoptotic pathways. Multiple targets- and receptor-coupled signal transduction can directly or indirectly modulates the nuclear transcription factors NF-κB, NFATc1, STAT3, and HIF-1α, which in turn regulate the production of several downstream pro-inflammatory cytokines, chemokines, immunocytes maturation and differentiation, immune complexes, proliferation, and apoptosis regulatory genes. Among these NP&TDs, the tripterygium-type ingredients, the artemisinin-type ingredients, and the paeony-type ingredients have been reported to be the mainstay in treating RA. Mechanistically, immunosuppression and anti-inflammation are still the primary therapeutic mechanisms. Nevertheless, the direct binding targets and pharmacodynamic mechanisms require further in-depth studies.

## 1 Introduction

Rheumatoid arthritis (RA) is a long-term autoimmune inflammatory disease distinguished by the involvement of multiple joints triggered by a combination of genetic, hormonal, and environmental components (1). Its clinical manifestations are symmetrical polyarticular swelling and pain, deformity, and movement disorders, frequently involving the small joints of the extremities accompanied by morning stiffness (2). The pathological process of RA involves aggressive synovial inflammation in multiple joint cavities, formation of vascular opacities, and progressing damage to local cartilage (3). Pro-inflammatory cytokines and chemokines produced by synovial cells and infiltrating immune cells are directly correlated with the occurrence and development of RA (4). The excessive secretion of these pathogenic factors subsequently results in the release of substantial quantities of inflammatory mediators, which exacerbate damage to the synovial membrane and cartilage within the joint, ultimately leading to swelling and stiffness at the affected joint. Macrophages and fibroblast-like synoviocytes (FLS) represent two pivotal cell types implicated in the pathogenesis of RA. Upon the onset of local tissue inflammation, macrophages contribute to synovial and cartilage tissue damage through the secretion of various pro-inflammatory cytokines. Concurrently, FLS play multifaceted roles in RA progression, including the promotion of inflammatory responses and facilitation of joint invasion. The pathological hyperactivation and proliferation of these two cell populations are considered central to the development and progression of RA (5, 6).

There is no complete clinical cure for RA, but it can be effectively controlled with medications. Clinically, the drugs used to treat RA mainly include non-steroidal anti-inflammatory drugs (NSAIDs), disease-modifying anti-rheumatic drugs (DMARDs), glucocorticoids, and immunosuppressants (7, 8). These therapeutic agents demonstrate efficacy in alleviating clinical symptoms and mitigating localized tissue erosive lesions in patients. However, their long-term administration may induce immunosuppression and lead to adverse effects, including pneumonia, urinary tract infections, and oral ulcerations, thereby limiting their clinical applicability. In contrast, certain NP&TDs, such as tripterygium-like drugs, artemisinin-like drugs, and sinomenine, have shown promising therapeutic outcomes in reducing joint swelling and morning stiffness while offering the advantages of reduced adverse effects and lower treatment costs (9–11). Therefore, they have been widely used in Chinese medicine clinics, but the active ingredient and the pharmacological mechanisms by which these NP&TDs have not yet been recognized as clear insights. Subsequently, it is of principal physiological significance to investigate the efficacy, target, and mechanism of NP&TDs in RA.

Generally, most NP&TDs are separated and extracted from natural plants and characterized by their complex chemical compositions and multifaceted pharmacological activities. Optimizing the chemical structure of NP&TDs, developing indications, and exploring various pharmacological mechanisms are of far-reaching significance. For decades, preclinical research has demonstrated that some botanicals have promising curative effects on RA, such as tripterygium, artemisinin derivatives, and total glucosides of paeony (12–14). However, a systematic review of this section is still absent. Thus, this review centers on summarizing the targets and mechanisms of NP&TDs against RA in preclinical experimental studies.

## 2 Methods

The principle of this review’s search for relevant literature was to screen the search tool (PubMed MEDLINE, Web of Science) for publications associated with the targets and mechanisms of NP&TDs against RA by March 5, 2025. The literature screened was kept within five years of publication as far as possible. The keywords used in the search include the following vocabulary: “NP&TDs against rheumatoid arthritis”. The NP&TDs covered in this paper were screened from a wide range of ingredients that meet the following criteria that have been highlighted for research or report to have potential medicinal value. The exclusion criteria for searching the relevant literature for this review was not to consider trials of these herbal active ingredients or their combination with existing chemical substances. Another exclusion criterion was to not consider inflammatory responses caused by pathogenic microorganisms. This review provides comprehensive and systematic preclinical experimental research on the targets and mechanisms of active components against RA and provides a theoretical basis for conducting relevant clinical trials to demonstrate the therapeutic potential of active components in RA. On March 12, 2025, plant names in the paper were verified and rectified through the “Plant List” website (http://www.theplantlist.org). Relevant mechanism diagrams are drawn by the “Figdraw 2.0” website.

## 3 Rheumatoid arthritis

### 3.1 Terpenoid

#### 3.1.1 Triptolide

Triptolide (TP) is the primary effective ingredient of tripterygium-like active ingredients in relieving RA symptoms. TP has various biological functions, including immunosuppression, chondroprotection, and anti-inflammatory, and has been shown to treat certain inflammatory and autoimmune disorders (15). In *in vivo* studies, it was reported that the typical signaling of TP primarily centers on cytokines and cellular immune signaling, such as TREM-1 signaling. It is worth mentioning that TP can reduce the expression of TREM-1 and DAP12 and suppress the revitalization of the JAK2/STAT3 signaling in the CIA rats, thereby effectively reducing inflammatory cytokines and chemokines in the serum and joint, such as TNF-α, IL-1β, and IL-6 (16). It also was reported that TP ameliorates joint symptoms in rats with collagen-induced arthritis by inhibiting URI1 and catenin signaling and decreasing RA-FLS hyperactivation response (17). TP inhibits Th17 differentiation by controlling PKM2-mediated glycolysis, thereby ameliorating joint inflammation in CIA mice (18). In *in vitro* studies, TP promotes chondrocyte proliferation and secretion through the down-regulation of miR-221 in exosomes of RA-FLS (19). Furthermore, TP may also down-regulate the expression of lncRNA ENST0000619282, thereby promoting the apoptosis of RA-FLS and ultimately improving inflammation (20). TP suppresses the inflammatory response in RA-FLS by regulating the has-circ-0003353s/microRNA-31-5p/CDK1 signaling axis (21). TP modulates neutrophil function through the Hippo signaling pathway to alleviate pathological damage in RA (22). Bioinformatics analysis showed that the gene expression of IGF2BP3 was increased in RA synovial tissue, while TP can decrease the expression of IGF2BP3 in human PBMC and MH7A, so IGF2BP3 may serve as a prospective target for the therapeutic effect of TP on RA (23). Overall, TP has been widely studied for the treatment of RA and has been clinically used as a major component in China. TP has been reported to exert its efficacy mainly by suppressing the hyperimmune and anti-inflammatory pathways. However, there is a lack of conclusive evidence to validate the direct target and mechanism of TP, and in-depth *in vitro* and *in vivo* experiments are needed to validate TP in the future and to provide constructive guidance for the rational use of the drug in the clinic.

#### 3.1.2 Celastrol

Celastrol is also one of the monomer active components extracted from Tripterygium, which has various biological activities and has been regarded as a valid activity component in the prevention of RA. In *in vivo* studies, it has been reported that celastrol induces autophagy by the inhibition of the PI3K/AKT/mTOR signaling pathway to ameliorate joint inflammation in CIA mice (24). In *in vitro* studies, celastrol regulates Hsp90-NLRP3 interaction and inhibits synoviocyte proliferation and migration, thereby alleviating the pathological process of RA (25). In addition, celastrol can downregulate the transcription of certain inflammatory factor genes in RA by inhibiting the activity of Ca^2+^-ATPase in the endoplasmic reticulum and promoting Ca^2+^-mediated death of FLS (26). In nanomedicine, inflammatory targeted celastrol nanomedicine attenuates collagen-induced joint inflammation phenotype through NF-κB and Notch1 pathways (27). Moreover, targeted delivery of celastrol-loaded nanoparticles to the finger joint section effectively attenuates the inflammatory response of RAW264.7 cells and MH7A cells and joint symptoms in CIA rats via the ROS/NF-κB inflammasome axis (28). In general, celastrol is a widely studied natural product active ingredient for the treatment of RA, and it ameliorates the joint inflammatory phenotype of RA primarily through an anti-inflammatory pathway. However, the direct targets of TP anti-inflammatory action and effector target cells have not been fully elucidated and need to be explored in in-depth preclinical and clinical trials to provide an experimental basis for clinical practice.

#### 3.1.3 LLDT-8

Given that TP has a certain degree of hepatotoxicity and cytotoxicity, its application in clinical practice is restricted. Interestingly, LLDT-8 is a low-poisonous TP derivative that has been chemically optimized and shown to have excellent therapeutic impacts on RA. In *in vivo* studies, LLDT-8 regulates RANK and its ligand RANKL, thereby reducing osteoclast production and increasing osteoprotegerin levels in peripheral blood and synovial fluid. It also promotes the excretion of some inflammatory factors, thereby alleviating the inflammatory response (29, 30). In *in vitro* studies, LLDT-8 could target activation of downstream E2F1 and p53 signaling through modulation of the lncRNA-WAKMAR2/miRNA-4478 axis, thereby suppressing proliferation, migration, and invasion of RA-FLS (31). Bioinformatics research revealed that LLDT-8 primarily regulates the systemic and specific immune-related pathways of RA-FLS or targets inhibition of the T-cell surface antigen CD2 to perform its potent anti-inflammatory and immunosuppressive function in RA (32, 33).

**Figure 1:**
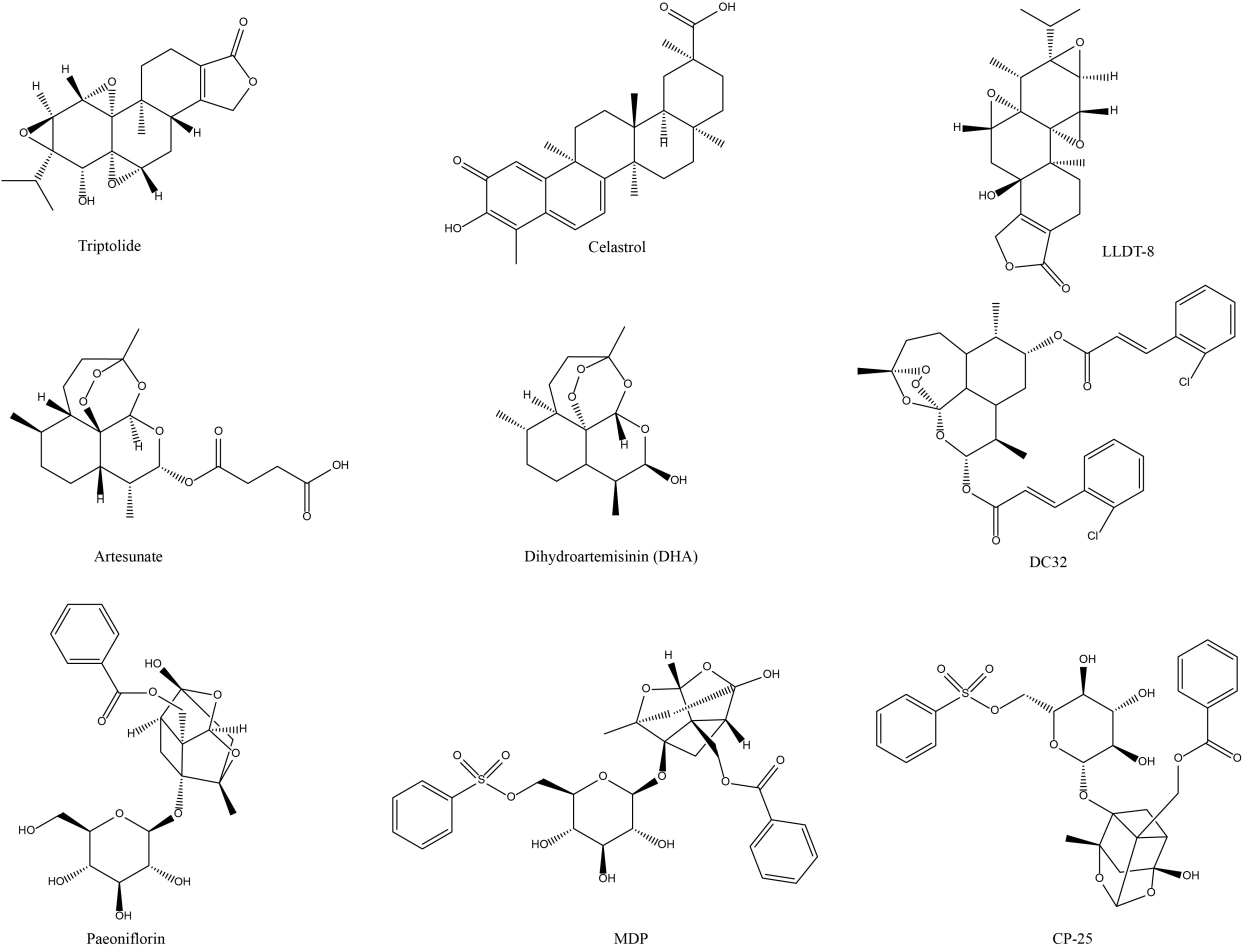
Chemical structures of the terpene active components of NP&TDs with mitigating effects on RA.

**Figure 2:**
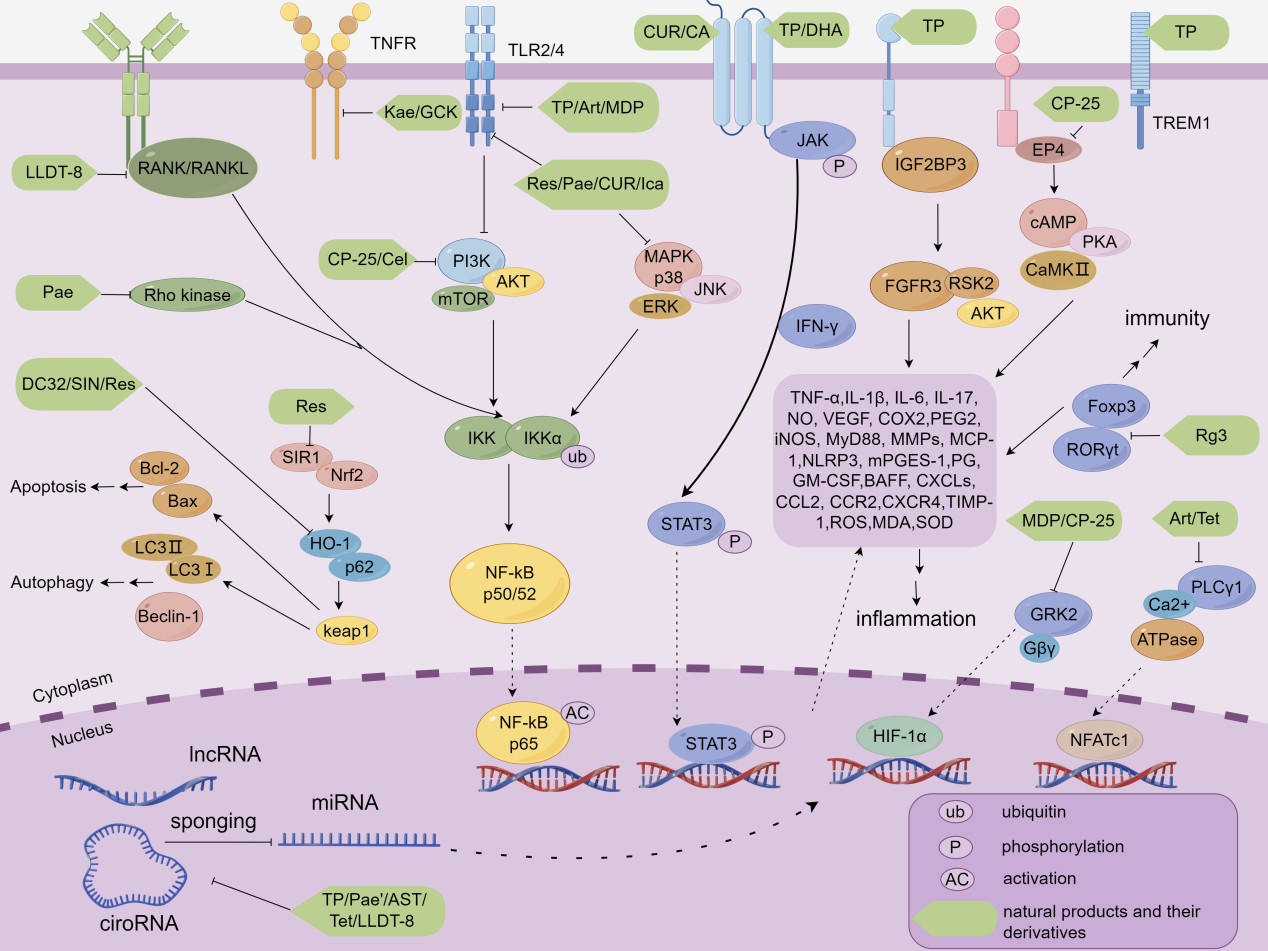
Molecular mechanisms by which NP&TDs mitigate RA through inflammatory, immune, and autophagic pathways. TP, Triptolide; Celastrol, Cel; LLDT-8, TP derivative; Art, Artesunate; DHA, Dihydroartemisinin; DC32, artemisinin derivative; Pae, Paeoniflorin; MDP, Paeoniflorin derivative; CP-25, Paeoniflorin derivative; AST, Astragaloside IV; GCK, Ginsenoside metabolite compound K; Rg3, Ginsenosides Rg3; SIN, Sinomenine; Tet, Tetrandrine; Res, Resveratrol; 1Pae’, Paeonol; CUR, Curcumin; Kae, Kaempferol; Ica, Icariin; CA, Curculoside A.

In the field of tripterygium-like active components therapy for RA, three active components of the research focus are TP, LLDT-8, and celastrol (**Figure 1**). All of them have potent immunosuppressive and anti-inflammatory activities. The promising progress has been made in terms of efficacy and mechanisms at the cellular and animal levels. Broadly speaking, in the *in vivo* phenotypic evaluation, these components improve the typical symptoms of RA to some extent, including joint inflammation, foot and claw swelling, behavioral-motor abnormalities, cartilage, and bone erosion. At the microscopic cellular level, they can restore the homeostatic imbalance between Treg/Th17 cells and maintain the balance between these two cell types to facilitate joint healing and disease treatment in RA. In terms of molecular mechanism exploration, a large variety of results provided pertinent evidence on the biological mechanisms and signaling pathways of these components against RA. These NP&TDs suppressed immune- or inflammation-associated signaling, such as transduction, phosphorylation, and nuclear translocation of multiple signaling molecules. In addition, other transcription factors, oxidative stress, DNA damage, and synovial fibroblast proliferation and migration are also implicated. Modulation of the biological processes of these molecular signals and consequent diminution of their downstream content of pro-inflammatory cytokines and chemokines (reduced production or accelerated degradation and emission) (**Table 1**, **Figure 2**).

**Table 1:** Therapeutic effects of natural products active components on RA.

Compound	Model		Mode of action	Reference
Triptolide (TP)	*In vivo*	CIA rats	Inflammation of cartilage and joints↓,TREM-1↓,DAP12↓,JAK2/STAT3 signaling pathway↓, TNF-α↓,IL-1β↓, and IL-6↓	(16)
		CIA rats	URI1↓, catenin↓	(17)
		CIA mice	Th17↓,JNK/MAPK↓, PKM2↓	(18)
	*In vitro*	RA-FLS	miR-221↓,TLR4/MyD88↓	(19)
		LPS-induced U937 histiocytic lymphoma cells	TREM-1↓, Inflammatory cytokines↓, IL-1β↓, and IL-6↓	(16)
		RA-FLS	lncRNA ENST0000619282↓	(20)
		RA-FLS	hsa-circ-0003353/microRNA-31-5p/CDK1↓	(21)
		Neutrophil	Hippo↓	(22)
		PBMC, MH7A	IGF2BP3↓	(23)
Celastrol	*In vivo*	CIA mice	PI3K/AKT/mTOR↓, autophagy↑	(24)
		CIA rats	joint inflammation↓, NF-κB↓, Notch1↓	(27)
		CIA rats	joint inflammation↓, NF-κB↓, ROS↓,TNFα↓,IL-1β↓,IL-6↓	(28)
	*In vitro*	RA-FLS	Hsp90-NLRP3↓, proliferation and migration↓	(25)
		RA-FLS	Ca^2+^-ATPase↓, pro-inflammatory cytokines and inflammatory factor genes↓	(26)
		RAW264.7 cell, MH7A cell	NF-κB↓, ROS↓,TNFα↓,IL-1β↓,IL-6↓	(28)
LLDT-8 (TP derivative)	*In vivo*	CIA rats	RANK↓, RANKL↓,OPG↑	(29, 30)
	*In vitro*	RA-FLS	lncRNA-WAKMAR2/miRNA-4478 axis↑, E2F1↑, p53↑	(31)
		RA-FLS	systemic and specific immune-related pathways↓, CD2↓	(32, 33)
Artesunate	*In vivo*	AA rats	NF-κB↓,HIF-1α↓, ROS↓	(34)
		CIA rats	p62↑, Nrf2↑,HO-1↑, NQO1↑, ROS↓,NFATc1↓,HO-1,NQO1	(35)
		AA rats	TLR4/TRAF6↓, PLCγ1-Ca^2+^–NFATc1 signaling pathway↓, Proliferation of osteoclastogenesis↓	(36)
	*In vitro*	RA-FLS	phosphorylation of AKT and RSK2↓, MMP-2↓,MMP-9↓, migration and invasion↓	(37)
		RAW 264.7 cells	NF-κB↓,HIF-1α↓, ROS↓, inflammation↓	(38)
Dihydroartemisinin (DHA)	*In vivo*	CIA mice	JAK/STAT3 signaling↓, NLRP3 inflammasome↓, HIF-1α↓, IL-1β↓,IL-6↓, inflammation↓, arthritic edema↓, cartilage destruction↓	(39)
DC32 (artemisinin derivative)	*In vivo*	CIA mice	Nrf2/HO-1/p62/Keap1 signaling↑, degradation of Keap1 protein↑, inflammation↓, cartilage degradation↓	(40)
		CIA mice	Treg/Th17 balance↑, IL-6↓, inflammation↓	(41)
	*In vitro*	NIH-3T3 cells	Akt/mTOR↓, ERK↓, Nrf2↑, degradation of Keap1 protein↑, inflammation↓	(40)
Paeoniflorin	*In vivo*	AA rats	IL-1β↓, IL-1↓, PGE2↓, IL-6↓, VEGF↓, GM-CSF↓, NF-κB p65↓, TNF-α↓, COX-2↓	(42)
		CIA rats	Rho kinase activity↓, NF-κB p65↓	(43)
		CIA rats	LIFR↓, aspirin↓	(44)
	*In vitro*	RA-FLS	CircRNA-FAM120A/miRNA-671-5p/MDM4 axis↓, proliferation↓, invasion↓, inflammation↓	(45)
MDP (Paeoniflorin derivative)	*In vivo*	AA in rats	p-GRK2↓, HIF-1α↓, NLRP3 inflammasomes↓	(46)
		AA in rats	TLR4/NLRP3/GSDMD signaling pathway↓	(47)
CP-25 (Paeoniflorin derivative)	*In vivo*	CIA rats	GRK2 translocation to EP4↓, GRK2 activity↓, GRK2 intracellular stabilization↑	(48)
		AA rats	Ahr activation↓, Ahr and GRK2 interaction↓	(49)
		Endothelial cells in AA rats	GRK2-stimulated CXCR4/ERK signaling↓, inflammatory angiogenesis↓	(50)
		Splenic T cells of AA rats	GRK2↓, EP4↓	(51)
		AA in rats	PGE2/EP4/cAMP signaling pathway↓	(52)
	*In vitro*	RA-FLS	GRK2 and Gβγ interaction↓	(53)
		RA-FLS	CXCR4/Gβγ/PI3K/AKT signaling↓, GRK2 translocation↓	(54)
Astragaloside IV (AST)	*In vivo*	AA in rats	IL-1β↓, TNF-α↓, NO↓, joint inflammation↓, joint swelling↓, chondrocyte proliferation↓	(55)
	*In vitro*	RA-FLS	lncRNA LOc100912373- miR-17-5p axis↓, PDK1↓, p-AKT↓, proliferation of FLS↓	(56)
		RANKL-induced osteoblasts	ERK signaling pathway↓	(57)
Ginsenoside metabolite compound K (GCK)	*In vitro*	RAW 264.7 cells/HUVEC cells	proliferation↓, apoptosis↓, immune responses↓CD8^+^ T cell↑, CD4^+^ T cells↓, M1-macrophages↓, TNF-α↓, IL-6↓	(58)
	*In vivo*	AA rats	TNF-α↓, TNFR2↓, joint swelling↓, pathological damage↓, proliferation and migration of RA-FLS↓	(59)
Ginsenosides Rg3	Freund’s adjuvant-induced mice		CD4^+^CD25^+^Foxp3^+^ Treg cells↑, inflammation↓, immunosuppressive↑	(60)
Sinomenine (SIN)	*In vivo*	CIA mice	IL-6↓, IL-12↓, IL-1α↓, TNF-α↓, IL-1β↓, CXCL1↓, IL-10↓, MCP-1↓, inflammation↓	(61)
		AA rats/FLSs	adenosine A_2_A receptor↑, NF-κB↓, TNF-α↓, inflammation↓, toe swelling↓, MCP-1↓, IL-6↓, VEGF↓	(62)
		CIA mice/FLS	phosphorylation of p62^Ser349^ and p62^Thr269/Ser272^↑,Keap1-Nrf2 signaling↑, IL-6↓, IL-33↓, ROS↓	(63)
		CIA mice	DNA-binding capacity of NF-κB↓, mPGES-1↓, PG↓	(64)
Tetrandrine	*In vivo*	AA in mice	neutrophil activity↓, IL-6↓, ankle swelling↓	(65)
		CIA in rats	PLCγ2-Ca^2+^ signaling↓, tyrosine kinase activity↓, NF-kB↓, NFATc1↓, osteoclastogenesis↓	(66)
	*In vitro*	RA-FLS	NEAT1/miR-17-5p/STAT3 signaling↓	(67)
Resveratrol	*In vivo*	AA rats	STAT3/HIF-1/VEGF pathway↓, Angiogenesis↓, inflammation↓	(68)
		AA rats	inflammation↓, COX-2↓, p-NF-κB↓, IL-1β↓, MMP-3↓, SIRT1-Nrf2 signaling↓	(69)
		CIA mice	inflammation↓,TLR4↓	(70)
		CIA rats	inflammation↓, ROS↓, HIF-1α↓, MAPK↓, p-JUN ↓	(71)
	*In vitro*	HUVEC cells	SIRT1 signaling↓, glycolysis↓, angiogenesis↓	(72)
		RA-FLS	serine-threonine kinase-p53 axis↓,apoptosis↑,cell cycle arrest↑	(73)
		RA-FLS	NF-κB ↓,proliferation↓, migration↓, apoptosis↑, SIRT1/Nrf2/HO-1↑, Keap1↓, cullin3↓	(74)
Paeonol	*In vitro*	TNF-α-mediated FLS	miR-155↓, FOXO3↑, IL-6↓, IL-1β↓, FLS proliferation↓	(75)
		IL-1β-induced RA-FLS	TNF-α↓, IL-6↓, IL-1β↓, MMPs↓, inflammation↓,TLR4-NF-κB signaling↓	(76)
	*In vivo*	CIA mice	TLR4↓, NF-κB↓, inflammation↓	(76)
Curcumin	*In vitro*	IL-1β-induced chondrocytes	Autophagy↑, NF-κB signaling pathway↓, apoptosis↑	(77)
		RA-FLS	JAK2/STAT3 signaling↓, linc00052/miR-126-5p/STAT2 axis↓, apoptosis↑, growth, migration and invasion of RA-FLS↓, inflammatory cell infiltration↓	(78)
		Macrophage	ROS↓, M2 macrophage↑, osteoblast↑	(79)
	*In vivo*	AIA rats	IκBα↓, COX-2↓, p-NF-κB/NF-κB↓	(80)
		CIA rats	TNF-α↓, IL-6↓, IL-1β↓, inflammation↓	(81)
Kaempferol	*In vitro*	RA-FLS	FGFR3-RSK2 signaling axis↓, IL-1β↓, IL-6↓, TNF-α↓	(82, 83)
		RA-FLS	migration of RA-FLS↓, TNF signaling pathway↓, AKT1↓, P-AKT1↓, JUN↓, P-JUN↓, CASP3↓, TNFR1↓,TNFR2↓	(84)
Icariin	*In vitro*	H-FLS	mitochondrial membrane potential↓, cytochrome C↑, ROS↑, migration and proliferation of FLS↓, cell cycle arrest↑, apoptosis↑	(85)
		RA- FLS	Nrf2↓, TRIB1↓, TLR2/NF-κB signaling↓, miR-223-3p/NLRP3 axis↓	(86, 87)
		RA-FLS	Proliferation↓, inflammation↓, GAREM1↓, MAPK↓	(88)
	*In vivo*	AA rats	ERK↓, HIF-1α↓, GLUT1↓, glycolysis↓, M1 macrophage↓, M2 macrophage↑	(89)
Curculoside A	*In vitro*	RAW 264.7 cells/THP1 cells	EGFR↓, MAP2K1↓, MMP2↓, FGFR1↓, MCL1↓	(90)
	*In vivo*	CIA rats	TNF-α↓, IL-1β↓, IL-6↓, IL-10↓, IL-12↓,IL-17A↓, joint swelling↓, arthritis scores↓, JAK/STAT/NF-κB signaling↓	(91)
	*In vivo*	AA rats	IL-6↓, IL-1β↓,PGE2↓,TNF-α↓,MDA↓, SOD activity↑, NF-κB signaling↓,NLRP3 inflammasome↓	(92)

The symbol “↑” represents that the signaling molecule (or symptom) is up-regulated (exacerbated), and the symbol “↓” represents that the signaling molecule (or symptom) is down-regulated (mitigated).

The numerous published perspectives together reveal an interesting phenomenon that TP, celastrol, and LLDT-8 can exert efficacy in RA-related cellular and animal scenarios. Personally, these ingredients hold great promise for future translational clinical applications, especially TP and celastro. They are more therapeutically effective and better studied. However, none of these studies have examined the toxic doses and adverse effects of the drugs in detail. Meanwhile, there is a lack of sufficient evidence for a more in-depth and detailed mechanism of action.

#### 3.1.4 Artesunate

In recent years, artesunate, dihydroartemisinin (DHA), and artemether have been reported to have great therapeutic potential in joint inflammation and autoimmune disorders (9). Artesunate is a semi-synthetic derivative of artemisinin. In *in vivo* studies, artesunate modulates ROS clearance and macrophage repolarization by restoring synovial homeostasis to ameliorate joint inflammatory phenotype in AA rats (34). Artesunate ameliorates bone erosion in CIA rats by modulating p62/Nrf2 and NFATc1 signaling and reducing ROS production (35). Artesunate reduces LPS-induced osteoclastogenesis by inhibiting the TLR4/TRAF6 and PLCγ1-Ca^2+^-NFATc1 signaling pathways, thereby alleviating bone damage in RA (36). Furthermore, artesunate drop MMP-2 and MMP-9 activity in the absence of inflammation, which may be accomplished by obstructing PDK-1-induced phosphorylation of AKT and RSK2, ultimately impeding the migration and invasion of primary RA-FLS (37). ROS-responsive artesunate pro-drug micelles inhibit the HIF-1α/NF-κB cascade and promote ROS clearance and macrophage repolarization in RA, thereby alleviating the inflammatory response and cartilage damage in RA (38). Overall, artesunate does not appear to rely on anti-inflammatory properties for its efficacy. Its pathway of action is focused on reducing the invasiveness of synovial tissue, thereby protecting articular cartilage and bone structures. Therefore, artesunate may be indicated in the late stages of RA. However, the molecular regulatory mechanism of artesunate in reducing the invasiveness of synovial tissues needs to be further validated to provide a key basis for promoting the development of new drugs and clinical use of artesunate.

#### 3.1.5 Artemisinin’s derivatives (DHA, DC32)

DHA is the active metabolite of artemisinin and its derivatives. Growing evidence claims that it has a powerful anti-inflammatory activity, which may have a therapeutic effect on certain inflammatory and autoimmune disorders. In *in vivo* studies, DHA may attenuate inflammatory responses, arthritic edema, and cartilage degradation in CIA mice by suppressing the JAK-STAT3 signaling pathway and the assembly of the NLRP3 inflammasome. This dual mechanism likely contributes to reduced HIF-1α expression and decreased serum levels of pro-inflammatory cytokines (39). In addition, the DHA derivative DC32 promotes the degradation of Keap1 protein by activating the Nrf2/HO-1/p62/Keap1 signaling, eventually alleviating inflammation in the foot joints and cartilage degradation of CIA mice (40). Secondly, DC32 can also alleviate joint inflammation in CIA mice by restoring Th17/Treg balance and decreasing the production of IL-6 (41). In *in vitro* studies, DC32 also inhibits the invasion and migration of FLS by reducing the secretion and release of MMPs and related chemokines (41). In the context of autophagy, DC32 modulates autophagic activity by enhancing Nrf2 expression and accelerating Keap1 protein degradation, thereby suppressing the production of pro-inflammatory cytokines. This regulatory effect may be associated with concurrent inhibition of the AKT/mTOR signaling pathway and the activation of the ERK cascade (40).

In the exploration of anti-RA targets and mechanisms of artemisinin-like drugs, the NP&TDs in focus are artesunate, DHA, and DC32 (**Figure 1**). They have shown favorable efficacy in immune function modulation and anti-inflammation, and have achieved initial effectiveness in the clinical application of RA in China. The exploration of the mechanisms in preclinical trials is also being extensively studied in depth. Specifically, in the evaluation of *in vivo* phenotypes, these components prevented and alleviated to some extent some symptomatic phenotypes of RA, including inflammatory reactions in pathologically damaged areas, joint swelling in the extremities, cartilage, and bone damage (**Table 1**). At the microscopic cellular level, these monomeric components similarly restore the balance between immune cells, synovial cells and osteoclasts, and maintaining this homeostatic balance counteracts the inflammation and invasiveness in the impaired pathological regions associated with RA. In the process of exploring the relevant molecular mechanisms, a multitude of data analyzed the targets and related signaling pathways of these four active ingredients for the therapy of RA. For example the transduction, phosphorylation, and nuclear displacement of multiple signaling and transcription factors, in addition to apoptosis, autophagy, and oxidative stress effects. Modulation of these signals in turn decreases the expression of their downstream pro-inflammatory cytokines and chemokines, which include (**Table 1**, **Figure 2**).

In summary, the above observations collectively reveal that artesunate, DHA, and DC32 can be efficacious in both cellular and animal experiments related to RA. Individually, artesunate has demonstrated more significant therapeutic effects, has been studied more extensively and intensively, and is expected to achieve development as a new drug in translational clinical applications. However, none of these studies have detailed toxic doses and adverse effects of the drug. There is also a lack of conclusive evidence detailing the direct targets and mechanisms of action of artesunate, which means that more in-depth research could be done in this area.

#### 3.1.6 Paeoniflorin

Numerous studies have confirmed the broad anti-inflammatory and immunomodulatory impacts of paeoniflorin (93). In *in vivo* studies, paeoniflorin can mitigate the secondary inflammatory response in AA rats by controlling the abnormal proliferation of synovial cells and down-regulating the expression of IL-1β, IL-1, PGE2, IL-6, VEGF, and GM-CSF, nuclear factor-κB p65, TNF-α, G protein and COX-2 in synovial tissue (42). Moreover, paeoniflorin may target the suppression of Rho kinase activity in synovial tissues and down-regulate the expression of NF-κB p65 and the content of pro-inflammatory cytokines, thus effectively ameliorating the foot and joint symptoms in CIA rats (43). A quantitative proteomic analysis of synovial tissue from CIA rats revealed that the two key targets of paeoniflorin for RA treatment are LIFR and aspirin (44). In *in vitro* studies, paeoniflorin may also target the regulation of the CircRNA-FAM120A/miRNA-671-5p/MDM4 axis to suppress the proliferation, invasion, and inflammation of RA-FLS (45). In conclusion, paeoniflorin ameliorates the pathologic phenotype of RA by inhibiting the abnormal growth of synoviocytes and the inflammatory cascade. However, the targets and molecular mechanisms of paeoniflorin’s effects on these two pathways have not been fully elucidated, and therefore it is a valuable line of research to work on these two aspects.

#### 3.1.7 Paeoniflorin’s derivative (MDP)

It has been investigated that the MDP impacts the maturation and differentiation of NLRP3 inflammasomes by targeting the suppression of GRK2 phosphorylation and modulating HIF-1α, which in turn diminishes the proliferation of FLS in AA rats (46). Similarly, MDP attenuates secondary inflammation and pathological injury in AA rats, which may be associated with modulation of the TLR4/NLRP3/GSDMD signaling pathway and thus suppression of macrophage apoptosis (47). In summary, MDP is mainly involved in the inflammatory cascade response in RA by interfering with the maturation and differentiation of NLRP3 inflammatory vesicles, which in turn reduces the pathological damage under inflammatory stimulation.

#### 3.1.8 Paeoniflorin-6-oxo-benzenesulfonic acid (CP-25)

Numerous studies have illustrated the powerful anti-inflammatory and immunomodulatory activities of CP-25 in RA. In *in vivo* studies, CP-25 exerts its therapeutic effects in experimental arthritis by directly targeting GRK2 through multiple mechanisms (1): enhancing GRK2 protein stability while suppressing its enzymatic activity via inhibiting EP4 receptor translocation (48); (2) disrupting GRK2-Gβγ interactions to restore EP4 receptor responsiveness (53); (3) blocking Ahr activation and its association with GRK2 (49); (4) attenuating CXCR4/Gβγ/PI3K/AKT signaling through GRK2 translocation blockade (54). Collectively, these actions inhibit FLS hyperproliferation and alleviate RA-associated inflammatory response and pathological process. In addition, CP-25 suppressed inflammatory angiogenesis around RA articular cartilage by downmodulating GRK2-stimulated CXCR4/ERK signaling in endothelial cells in AA rats (50). In *in vitro* studies, CP-25 also deregulated the expression of GRK2 and EP4 in splenic T cells, thereby impacting the functionalities of splenic T cells to exert anti-inflammatory and immunomodulatory effects (51). Activation of PGE2/EP4/cAMP signaling can trigger abnormal hyperfunction of dendritic cells, and CP-25 can interfere with this signaling pathway to inhibit the maturation and abnormal activation of dendritic cells (52).

In the area of total glucosides of paeony-targeted therapy for RA, the major active components examined are paeoniflorin, MDP, and CP-25 (**Figure 1**), which have potent immunomodulatory and anti-inflammatory effects and have made excellent headway in preclinical trials regarding efficacy and mechanism, promising the development of natural drugs for the clinical management of RA. In the evaluation of RA phenotypes, paeoniflorin and its derivatives were able to alleviate joint inflammation, swelling of foot joints and pedicles, bone erosion, cartilage destruction, and inflammatory angiogenesis around articular cartilage in experimental arthritis. Regarding microscopic cellular interactions, paeoniflorin and its derivatives can adjust the function and proliferation of immune cells (T lymphocytes, B lymphocytes, Treg, Th17, macrophages, and dendritic cells) associated with the pathological zone of joint inflammation. It also decreases the infiltration of aggressive cells in the pathological regions, the proliferation, migration, and invasion of synovial cells, and the production and activation of osteoclasts, thus diminishing the generation of pro-inflammatory cytokines and chemokines, and the repair of damage to joints and cartilage to some extent. In the microenvironment of signaling molecular interactions, a large body of published data provides information on the biological mechanisms of paeoniflorin and its derivatives to alleviate the occurrence and development of RA. Overall, they suppress the transmission of signaling pathways associated with immunity or inflammation, such as transduction, phosphorylation, interactions, translocation, and regulation at the gene level between multiple signaling factors, in addition to other transcription factors, such as HIF-α, cell differentiation, apoptosis pathways, and DNA damage. By moderating the signaling behavior of these molecules and thereby decreasing the levels of their downstream RA-related inflammatory mediators and autoantibodies (**Table 1**, **Figure 2**).

### 3.2 Saponins

#### 3.2.1 Astragaloside IV

Astragaloside IV (AST) is an organic compound extracted from *Astragalus membranaceus (Fisch.) Bunge* of leguminous plants, which has anti-inflammatory, antioxidant, anti-apoptosis, progressing cardiopulmonary function, adjusting abnormal immune performance, and other biological effects (94). However, its current research on RA is not sufficient. In *in vivo* studies, AST ameliorated joint inflammation in AA rats, diminished the levels of IL-1β, TNF-α, and NO, and improved IL-1β-induced chondrocyte proliferation and joint swelling (55). In *in vitro* studies, AST may also downgrade the expression of PDK-1 and p-AKT via regulating the lncRNAloc100912373/miR-17-5p axis, subsequently turning around the overproliferation of RA-FLS (56). Moreover, AST also reduced RANKL-induced osteoclastogenesis by repressing the ERK signaling pathway (57).

**Figure 3:**
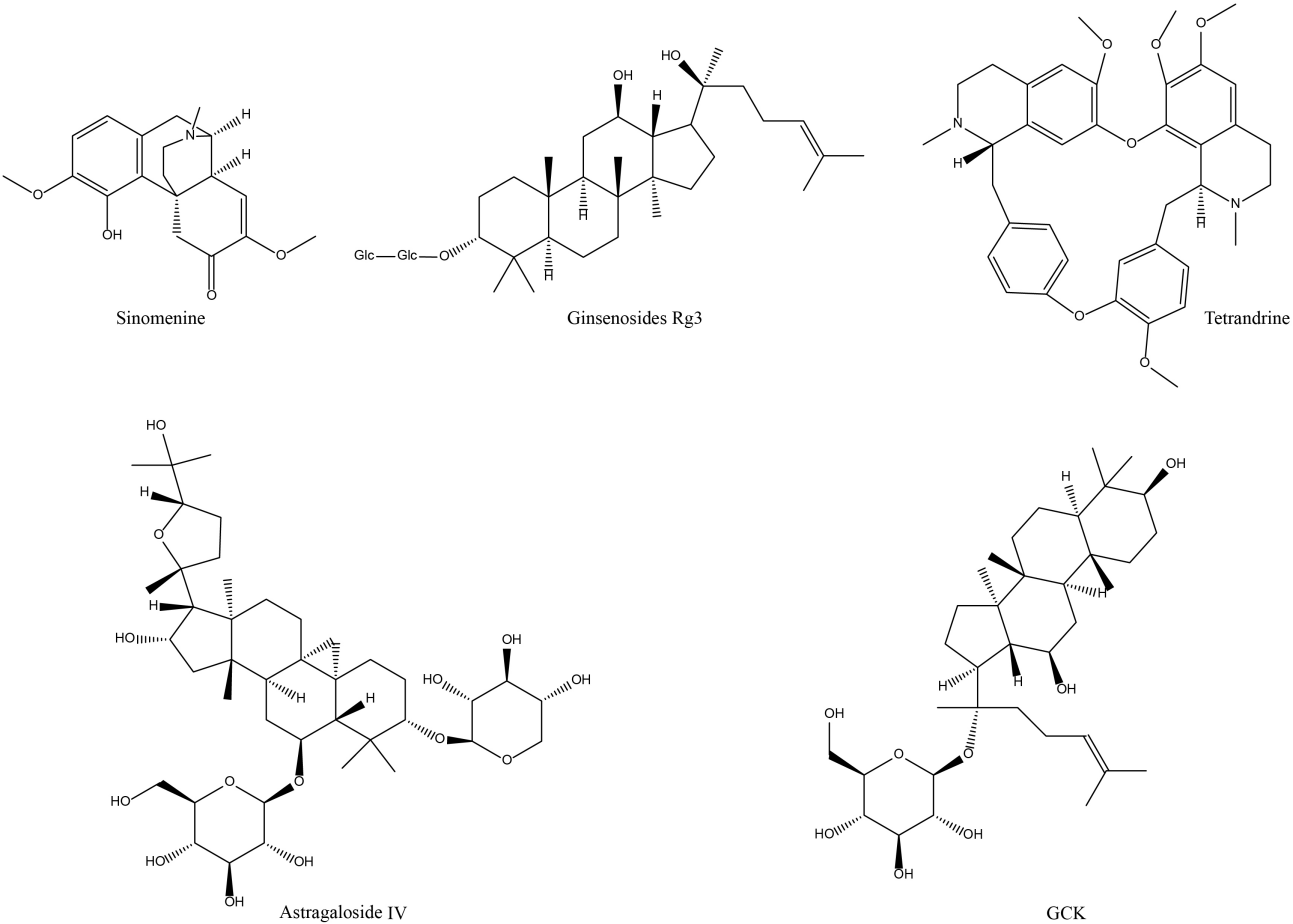
Chemical structures of the polyphenols and flavonoids active components of NP&TDs with alleviating effects on RA.

In a nutshell, AST (**Figure 3**) is mainly exploited in the therapeutic mechanism of experimental RA to modulate the immune and anti-inflammatory processes, attenuate some of the symptoms of experimental joint inflammation and cartilage erosion, regulate the transmission of relevant signaling pathways, the expression of gene signals and the function of osteoclasts, and down-regulate the expression of inflammation-related factors (**Table 1**, **Figure 2**). Nevertheless, this part of the research has remained extremely shallow, and the therapeutic effect on RA still needs to be examined. Clinically, AST is more commonly used to enhance the body’s immune function, which is contrary to the pathological microenvironment of RA with over-activated immune cells. Therefore, I personally consider that AST is unsuitable for the treatment of clinical RA.

#### 3.2.2 Ginsenoside metabolite compound K and Ginsenoside Rg3

Ginsenosides are steroid compounds extracted from the rhizomes of the Araliaceae plant ginseng. About 40 kinds of monomers have been identified, including ginsenosides Rg1, Rg2, Rg3, Rb1, Rh2, Rc, Rd, and Re. It has various pharmacological effects such as protecting cardiovascular and cerebrovascular disorders, anti-inflammatory, anti-tumor, improving learning and memory, and regulating immune system function (95). Among them, the research on RA is mainly about GCK. In *in vitro* studies, GCK can inhibit the proliferation and apoptosis of LPS-induced RAW 264.7 cells and TNF-α-induced HUVEC cells (58). On the other hand, GCK augments CD8^+^ T cell populations to suppress excessive immune activation, concurrently reducing activated CD4^+^ T cells and pro-inflammatory M1-polarized macrophages, leading to decreased TNF-α and IL-6 production (58). In *in vivo* studies, GCK targets RA-associated inflammation by regulating TNF-α/TNFR2 signaling in synovial cells and significantly alleviates joint pathology in AA rats, including attenuation of synovial hyperplasia and inhibition of RA-FLS proliferation/migration (59). In addition to GCK, Ginsenoside Rg3 has anti-inflammatory and immunosuppressive effects on RA mice. Its mechanism may be related to regulating the oxidative phosphorylation pathway and enhancing the function of CD4^+^CD25^+^Foxp3^+^ Treg cells to maintain peripheral immune tolerance in RA mice (60).

GCK is another saponin-like active component for the amelioration of RA (**Figure 3**), and several studies have investigated its role and mechanism in experimental RA models. The compound demonstrates significant biological efficacy through dual mechanisms: regulation of T lymphocyte differentiation and macrophage polarization, coupled with potent suppression of pro-inflammatory mediators. From macroscopic animal phenotypes, GCK ameliorates RA-related clinical manifestations, such as inflammatory responses and pathological damage at the associated joints and ankle swelling. GCK exerts its immunomodulatory effects through multiple mechanisms: it significantly enhances the differentiation and proliferation of CD8^+^ cytotoxic T cells while simultaneously suppressing the activation and expansion of CD4^+^ T helper cells. Concurrently, GCK modulates macrophage polarization by inhibiting the formation of pro-inflammatory M1 phenotype macrophages. These coordinated immunoregulatory actions effectively attenuate excessive immune responses. Additionally, GCK demonstrates remarkable therapeutic potential by inhibiting the pathological proliferation and migration of synovial fibroblasts, while suppressing osteoclastogenesis. This dual inhibition mechanism ultimately protects synovial membranes and cartilage from inflammatory mediator-induced erosion and degradation. Regarding mechanism exploration, GCK can adjust the TNF-α/NF-κB, JNK, and ERK signaling which are associated with RA. By regulating these signaling molecules, GCK hindered the pathogenic aggressive activity of downstream signaling molecules, such as MMPs, TNF-α, IL-6, and RANKL (**Table 1**, **Figure 2**). However, this part of the study lacks in-depth research on the specific mechanisms of immunomodulation. In addition, from the available evidence, the anti-inflammatory activity of GCK in the treatment of RA is not as favorable as that of other active ingredients, and it mainly exerts part of its effect through immune modulation, which means that GCK may not be able to produce better efficacy in middle- and late-stage RA.

### 3.3 Alkaloid

#### 3.3.1 Sinomenine

Sinomenine (SIN) is a kind of alkaloid isolated from the plant *Singophyllum sylvestris* with various biological functions such as analgesia, antitussive, hypotensive, anti-inflammatory, and immunomodulation (96–98). In *in vivo* studies, SIN can regulate the secretion of various inflammatory cytokines and monocyte/macrophage chemokines and subsequently alleviate the inflammatory storms in CIA mice (61). In addition, SIN increased the interaction of α7nAChR and adenosine A_2_A receptor and restricted NF-κB signaling in AA rats, thereby reducing arthritic inflammation, toe swelling, and serum TNF-α levels, while down-regulating the level of MCP-1, IL-6, and VEGF in LPS-induced FLSs (62). In RA-FLS, SIN may also activate Keap1/Nrf2 signaling by reversing the phosphorylation of p62^Ser349^ and p62^Thr269/Ser272^, subsequently lessening the secretion of IL-6 and ROS to improve the degree of articular cartilage damage (63). Moreover, SIN exerted its anti-inflammatory activity in CIA mice by downgrading the DNA-binding capacity of NF-κB to selectively inhibit mPGES-1 expression and prostaglandin overproduction (64).

Regrading alkaloid therapy for RA, the most investigated active monomeric component is SIN (**Figure 3**). SIN has initial effectiveness in the clinical practice of RA in China, and the exploration of pertinent mechanisms is continuing. In experimental RA models, SIN similarly alleviated RA-related symptom phenotypes to a certain extent, mainly in terms of inflammatory responses in pathologically damaged areas, and toe swelling (**Table 1**). In the cellular microenvironment, SIN regulates the frequency and transport of Treg/Th17 cells in lymphoid tissue, maintaining the balance between Treg/Th17 and limiting its transport to the joints contributes to controlling the development of RA. In terms of mechanistic investigations, numerous data have described molecular signaling regulatory mechanisms in experimental RA models treated with SIN. For example, the activation of multiple signaling pathways that mediate phosphorylation and nuclear displacement of different sites of proteins, in addition to processes involved in DNA transcriptional capacity, FLS invasive capacity, and oxidative stress. Regulation of these signals in turn diminishes the levels of their downstream inflammatory mediators (**Table 1**, **Figure 2**). In conclusion, the above findings jointly demonstrate that SIN can exert anti-RA efficacy in several aspects, especially in regulating immune cell function. However, the lack of conclusive evidence detailing SIN’s direct targets and mechanisms of action implies that further research is required in this field.

#### 3.3.2 Tetrandrine

Tetrandrine is an active component of alkaloids extracted from plants of the family Proteaceae with pharmacological activities, including antipyretic, analgesic, and anti-inflammatory (99). In *in vivo* studies, it has been investigated that tetrandrine may alleviate the degree of ankle swelling by suppressing neutrophil activity and IL-6 expression in AA mice (65). In addition, tetrandrine may also impede PLCγ2-Ca^2+^ signaling by blocking tyrosine kinase activity and the nuclear entry of two transcription factors (NF-kB and NFATc1), thereby attenuating RANKL-induced osteoclastogenesis during pathogenesis in CIA rats and preventing damage to articular cartilage and bone (66). In *in vitro* studies, tetrandrine could limit the overproliferation of RA-FLS and induce programmed cell death by impeding NEAT1/miR-17-5p/STAT3 signaling (67).

Although less widely studied and clinically applied than SIN, tetrandrine (**Figure 3**) has also been reported to have favorable effects in alleviating the development of RA. In an experimental arthritis model, tetrandrine partially reduced the degree of swelling and cartilage and bone damage at the joint, alleviated the aggressiveness of synovial cells, and reduced the secretion of inflammatory factors by macrophages and chondrocytes. These effects may be mediated through the inhibition of neutrophil and tyrosine kinase activity *in vivo*, blocking nuclear displacement of NF-kB and NFATc1, downregulation of migratory proteins, and inhibition of PI3K/Akt and JNK signaling (**Table 1**, **Figure 2**). Nevertheless, all of these mechanisms are broad and have not elucidated a detailed major pathway of action, nor have studies of their adverse effects been reported. Additionally, there is a lack of corresponding forward and reverse target mechanism validation experiments, and still a far distance from being translated into new clinical medicines.

### 3.4 Polyphenols

#### 3.4.1 Resveratrol

Resveratrol, a polyphenolic organic compound, is an antitoxin secreted by many plants when stimulated by foreign agents (100). Naturally occurring resveratrol has antioxidant, anti-inflammatory, anti-aging, and immunomodulatory biological activities, and has a promising therapeutic impact on many experimental RA models (101). In *in vivo* studies, resveratrol mitigates joint inflammation and pathological neovascularization by inhibiting the STAT3/HIF-1α/VEGF signaling pathway (68) or activating of SIRT1-Nrf2 signaling pathway (69). Resveratrol alleviates neutrophil extracellular traps in RA mice by inhibiting TLR4 mediated inflammatory responses (70). Resveratrol also inhibited the inflammatory response and ROS generation at the synovial tissues of CIA rats, reduced the aberrant expression of HIF-1α, MAPK, and p-JUN in IL-1β-stimulated rat synovial cells (71). At the cellular level, resveratrol was able to inhibit glycolysis-mediated aberrant angiogenesis by activating SIRT1 signaling in HUVEC cells (72). Furthermore, resveratrol promotes RA-FLS apoptosis and induces cell cycle arrest by regulating autophagy and the serine-threonine kinase-p53 axis (73). Resveratrol also inhibits NF-κB activation and RA-FLS proliferation and migration and induces apoptosis (74). These effects are potentially mediated through SIRT1/Nrf2/HO-1 signaling activation, which downregulates Keap1 and cullin3 expression.

**Figure 4:**
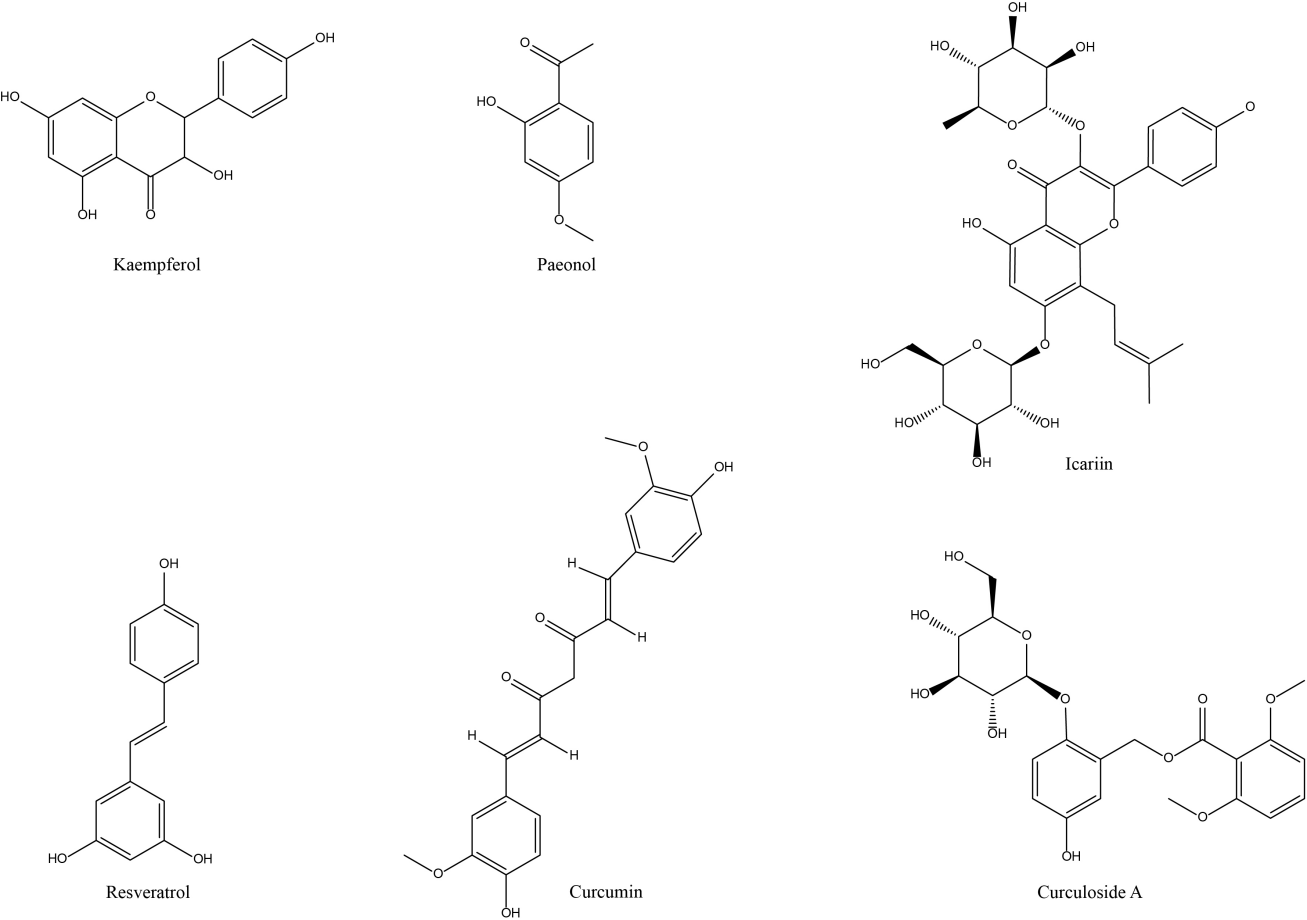
Chemical structures of the saponins and alkaloid active components of NP&TDs with alleviating effects on RA.

Overall, resveratrol (**Figure 4**) primarily modulates various inflammation-related signaling molecules, angiogenesis, and synovial functions to delay the progression of experimental arthritis (**Table 1**, **Figure 2**). In particular, it may exert a momentous role in regulating the function and expression of transcription factors Nrf2 and HIF-1α. However, there is a lack of corresponding mechanism verification and investigation of the function of systemic immune cells. From the existing evidence, resveratrol does play a partial role in RA models. However, due to its low relative bioavailability and rapid *in vivo* distribution and metabolism, it is difficult to apply its prototype components in clinical practice.

#### 3.4.2 Paeonol

Paeonol is a phenolic active ingredient isolated from the root bark of Ranunculaceae Peony flowers with various biological effects, including antipyretic, analgesic, anti-inflammatory, and inhibiting allergy (102). It has a favorable inhibitory effect on inflammatory reactions caused by a variety of causes. However, there are few studies on paeonol in RA. In *in vitro* studies, paeonol could activate its target FOXO3 by modulating miR-155, thereby curbing TNF-α-stimulated FLS proliferation and the production of some inflammatory factors (75). In addition, it has also been pointed out that paeonol pretreatment prominently suppressed IL-1β-induced inflammatory response of RA-FLs, and reduced the production of TNF-α, IL-6, IL-1β, and MMPs (76). In *in vivo* studies, paeonol treatment alleviated the indications of arthritis in CIA mice, the mechanism may be associated with the hindrance of the TLR4/NF-κB axis (76).

Among the phenolic active components for the management of RA, the primary emphasis of investigation is on paeonol and resveratrol (**Figure 4**). In the therapeutic context of experimental RA, paeonol is utilized mainly for its anti-inflammatory function, alleviating some phenotypes of joint inflammation, diminishing the conduction and expression of relevant inflammatory signaling pathways (TLR4/NF-κB, PI3K/AKT/NF-κB) and downregulating the levels of inflammation-related mediators (**Table 1**, **Figure 2**). It has been clinically used for pain and activity limitation caused by osteoarthritis and has a promising future clinical application for RA. However, the above studies lack the corresponding mechanism verification and related experiments on immune cells and associated experimental studies on toxicity and adverse reactions. They need a lot of preclinical and clinical practice before they can be used for the clinical treatment of RA.

#### 3.4.3 Curcumin

Curcumin (CUR) is a polyphenolic compound extracted from some plants of *Zingiberaceae* and *Araceae*. It has extensive biological activities, including anti-inflammatory, antioxidant, and anti-bacterial infection, lowering blood lipids, protecting the liver and gallbladder, protecting cardiovascular and regulating immune system function (103–105). CUR exhibits more prominent antioxidant and anti-inflammatory capabilities and can play a role in a variety of autoimmune diseases (106). However, its low bioavailability makes its anti-inflammatory activity *in vivo* and *in vitro* experiments vary broadly, so multiple high-dose oral doses are utilized in animal bioassays.

In *in vitro* studies, multiple high doses of oral CUR may ameliorate IL-1β-stimulated apoptosis in rat primary articular chondrocytes by regulating autophagy and inhibiting the NF-κB pathway (77). Moreover, CUR modulates the linc00052/miR-126-5p/PIAS2 axis and JAK2/STAT3 signaling, promotes RA-FLS apoptosis, inhibits its growth, migration, and invasion, and improves inflammatory cell infiltration, thus exerting its anti-RA activity (78). In nanomedicine, a copper silicate nanoparticle loaded with water-soluble Zn-CUR can be degraded in the acidic microenvironment of arthritic regions. It can effectively scavenge ROS from M1 macrophages, promote their conversion to an anti-inflammatory M2 phenotype, and facilitate osteoblast biotransformation (79). In *in vitro* studies, BDMC (a derivative of CUR) demonstrated significant efficacy in ameliorating hindfoot swelling, reducing arthritis indices, and attenuating arthropathological damage in rats. This was achieved by inhibiting the degradation of IκBα, downregulating COX-2 levels, decreasing the p-NF-κB/NF-κB ratio, and impeding macrophage migration (80). A ROS-responsive curcumin nanomicelle significantly inhibits inflammatory factor levels, prolongs drug circulation time, and prevents cartilage erosion (81).

As previously mentioned, CUR (**Figure 4**) is another phenolic active component for the treatment of RA. CUR is not yet available as a stand-alone drug for the clinical management of RA in China due to its bioavailability, but investigations of related mechanisms in preclinical RA trials have been reported. It is principally reflected in the fact that CUR alleviates to some extent the symptomatic phenotypes associated with RA, such as inflammatory response in the damaged region of the joint, swelling of the foot and claw, and cartilage and bone destruction. At the microscopic cellular level, CUR inhibits apoptosis and differentiation of chondrocytes as well as proliferation, migration, and invasion of synovial cells, contributing to the prevention of synovial and cartilage repair. In terms of mechanism exploration, CUR regulates molecular signaling mechanisms of inflammation in experimental RA models. It is also implicated in processes that autophagy, FLS invasion and apoptosis, inflammatory cell infiltration, and gut-associated factors (**Table 1**, **Figure 2**). In recent years, for the development of curcumin nanomedicine is a promising research idea. It is able to improve the bioavailability and therapeutic effect of curcumin by utilizing the targeting properties of nanomaterials. Overall, CUR has good prospects for clinical application in RA. However, there is a lack of validation of drug binding targets and mechanisms as well as studies on immune cell function in this area, and more in-depth practical studies are needed.

### 3.5 Flavonoids

#### 3.5.1 Kaempferol

It was also reported that 144 biologically active compounds were screened from Tripterygium, of which kaempferol is an important anti-RA active compound with suppresses inflammatory and overimmune (107). In particular, kaempferol can decrease serum levels of inflammatory factors IL-1β, IL-6, and TNF-α by modulating the FGFR3-RSK2 signaling axis (82, 83). Moreover, kaempferol may exert its therapeutic effect on RA by restricting the migration of RA-FLS and the TNF signaling and regulating the activation status of related biological targets (84). Overall, kaempferol plays a therapeutic role in RA mainly by inhibiting the inflammatory cascade. However, few studies in this area have been reported, and its direct targets, effector target cells and molecular regulatory mechanisms need to be further investigated.

#### 3.5.2 Icariin

Icariin, a flavonoid active component, is the main bioactive component of Epimedium, which has promising ameliorative effects in cardiovascular disorders (108) (**Figure 4**). It has gained attention in the treatment of RA due to its role in immunomodulation and bone metabolism. In *in vitro* studies, icariin decreased mitochondrial membrane potential and upregulated cytochrome C and ROS levels, thereby reducing the migration and proliferation of FLS and inducing its cell cycle arrest and apoptosis (85). Icariin also further ameliorated the inflammatory activation and hyperproliferative state of TNF-α-stimulated RA- FLS, which may be achieved by specifically upregulating the expression of Nrf2 and TRIB1 to suppress the over-activation of TLR2/NF-κB signaling pathway or by modulating the miR-223-3p/NLRP3 axis (86, 87). In addition, icariin also inhibits proliferation and inflammatory response of RA synoviocytes via GAREM1/MAPK signaling pathway (88). In *in vivo* studies, adipose-derived stem cell exosomes loaded with icariin alleviate inflammatory response and cartilage damage in RA by inhibiting the ERK/HIF-1α/GLUT1 pathway, reducing glycolysis, and promoting M1-M2 phenotypic transition (89). In conclusion, icariin mainly regulates bone metabolism and inflammation-related protein expression, the abnormal activation status of synovial cells, and invasive immune cells to facilitate local inflammatory response and cartilage damage in RA-related cellular and animal models (**Table 1**, **Figure 2**).

#### 3.5.3 Curculigoside A

CA is the main active compound extracted from *Curculigo rhizomes*, which has significant pharmacological impacts, including antioxidant, anti-osteoporosis, anti-depression, and neuron protection. In *in vitro* studies, CA pretreatment reduced the expression of EGFR, MAP2K1, MMP2, FGFR1, and MCL1 genes in an *in vitro* inflammatory cell model (90). In *in vivo* studies, CA alleviated paw swelling in CIA rats and lowered serum levels of pro-inflammatory cytokines and chemokines (TNF-α, IL-1β, IL-6, IL-10, IL-12, and IL-17A), the molecular mechanism of which may be relevant to the suppression of the JAK/STAT/NF-κB signaling pathway (91). Furthermore, CA prominently mitigated the foot and paw swelling in AA rats by a mechanism associated with inhibition of NF-κB signaling and NLRP3 inflammasome formation (92). CA primarily exploiting its antioxidant, anti-osteoporotic, and anti-inflammatory activities to mitigate some inflammatory symptoms and bone erosion in experimental RA models. (**Table 1**, **Figure 2**). Nevertheless, research in this area also lacks reports on the exact regulatory mechanisms and adverse effects of CA on bone erosion in late RA, and its anti-inflammatory effects are limited. Therefore, Therefore, CA is not yet directly translatable into clinical applications for the treatment of RA.

## 4 Discussion and prospect

With the increase in modern unhealthy lifestyles and cross-infection, the global prevalence of RA has shown a marked upward trend. This epidemiological shift poses significant clinical challenges, as delayed or inadequate treatment of these conditions may lead to severe pathological consequences and potentially life-threatening complications. However, most of the drugs used for the treatment of RA may produce a variety of complex adverse effects and multi-organ toxicity, limiting their clinical application to a certain extent. Therefore, many researchers have devoted themselves to the search for less toxic natural active compounds against rheumatism from natural plants and to explain their possible targets and mechanisms. In this paper, we focus on the therapeutic activity and potential pharmacological mechanisms of NP&TDs against RA. Overall, the bioactive mechanisms of NP&TDs described herein for treating these diseases include the involvement of multiple receptor-coupled signals, including JAK/STAT, PI3K/Akt, MAPK/ERK, Nrf2/HO-1, TREM-1, TLR4, JNK, PLC. The inhibition of these signals directly or indirectly impedes abnormal activation of the nuclear transcription factor NF-κB, which in turn regulates several downstream pro-inflammatory cytokines, inflammatory factors, chemokines, immunomodulatory receptors, immune complexes, and autoantibodies, such as TNF-α, IL-1β, IL-6, IL-8, IL-17, COX2, PEG2, VEGF, iNOS, MMPs, MCP-1, BAFF, IgG MyD88. In addition, the regulation of other nuclear transcription factors such as NFATc1 and HIF-α may also be involved.

Currently, modulation of systemic and local immune and inflammatory pathways remains the primary mechanism for most NP&TDs to alleviate the pathological process of RA. Partial studies have also addressed calcium signaling, oxidative stress, and autophagy pathways, and there is a necessary to explore more deeply the mechanisms and targets in this regard. In particular, the inhibition of “tumor-like” growth of fibroblastic synoviocytes, autophagy and apoptosis pathways deserve further investigation, as they are closely related to the important pathological and physiological processes involved in the progression of RA from early to late stages. Furthermore, the molecular mechanisms underlying RA pathogenesis, particularly the dynamic alterations in pathogenic gene expression throughout disease progression, remain insufficiently explored. Therefore, it is worthwhile to explore in greater depth the pathological mechanisms of key genes during RA development and the therapeutic mechanisms of NP&TDs. Recently years, the JAK/STAT signaling pathway has become a hot spot for RA research, and some JAK inhibitors have entered phase II and phase III clinical trials, such as some selective tinib-based JAK inhibitors. However, these immunosuppressants may be accompanied by sophisticated side effects caused by excessive immunosuppression, such as recurrent infections, fever, anemia, and bone marrow suppression. Therefore, screening for JAK inhibitors in active components with lower toxic effects is also an attractive research direction.

Numerous *in vivo* and *in vitro* experiments have confirmed the therapeutic effects of various NP&TDs on RA. A noteworthy issue is that if these of NP&TDs are used in clinical practice for the treatment of autoimmune diseases, it is likely that they will not be used as a single agent in the treatment of the disease. Instead, these NP&TDs may be used in combination therapies with other immunomodulators or anti-inflammatory agents to achieve enhanced efficacy and reduced toxicity, which raises the question of whether the interaction of two or more components is synergistic or antagonistic to the disease effect. For example, the combination of artesunate with immunomodulators could better promote the apoptosis of aggressive macrophages and improve immune function and inflammation in RA mice. However, the combination is not always beneficial, and toxic reactions may occur under specific pathological conditions, which need to be further explored in animal and clinical experiments. Another noteworthy issue is the safety and resistance evaluation of these NP&TDs, including the choice of the optimal dose of the drug, the mode of administration, and the timing of administration. Many novel compounds developed by pharmaceutical companies have been forced to terminate in clinical phase II or phase III trials because of unavoidable toxic effects. Although most of the animal data in the literature were conducted at non-toxic doses and no serious side effects were reported, cases of toxic reactions could not be completely avoided. Because the effective doses of these NP&TDs may be different for different species and diseases, and by the same token their safety ranges are also different, the effective and toxic dose ranges of drugs must be clarified before clinical trials.

The development of novel derivatives of some active components is an interesting research idea, and it is necessary to systematically explore the possibility of developing compounds with better activity and lower toxicity through medicinal chemical and structural modifications. It is also worthwhile to think about the effects of these NP&TDs on the action of immune cells and the regulation of immune function. Although the studies covered in this paper have exploited the immunosuppressive effects of NP&TDs, some studies have also reported the intervention of NP&TDs as immune enhancers for other diseases, such as cancer and immunodeficiency-like diseases. Thus, the positive and negative regulation of immune system function by drugs is also related to the specific pathological microenvironment of different cells and tissues in different disease types.

In conclusion, a large number of biological experiments have confirmed that NP&TDs exhibit unique excellence in autoimmune diseases and have potential clinical medicinal value. However, it is uncertain whether these active ingredients can be further developed into clinical drugs for autoimmune diseases, and more intensive preclinical and clinical trial studies are needed to translate the extensive preclinical results into clinical practice.

## Glossary

AA: adjuvant arthritis
Ahr: aryl hydrocarbon receptor
AKT: AKT serine/threonine kinase
AST: Astragaloside IV
BAFF: B cell-activating factor belonging to the TNF family
BAX: Bcl-2 associated X protein
Bcl-2: B-cell lymphoma-2
CA: Curculoside A
cAMP: cyclic adenosine monophosphate
CIA: type II collagen-induced arthritis
COX-2: cyclooxygenase-2
CP-25: paeoniflorin-6-oxo-benzenesulfonic acid
CUR: Curcumin
DHA: dihydroartemisinin
DMARDs: disease-modifying anti-rheumatic drugs
E2F1: E2F Transcription Factor 1
ECM: extracellular matrix protein
EGFR: epidermal growth factor receptor
EP4: E4 prostaglandin receptor
ERK: extracellular signal-regulated protein kinase
FGFR1: fibroblast growth factor receptor 1
FGFR3-RSK2: fibroblast growth factor receptor 3-ribosomal S6 kinase 2
FLS: fibroblast-like synovial cells
Foxp3: Forkhead box P3
GCK: ginsenoside metabolite compound K
GM-CSF: granulocyte-macrophage colony-stimulating factor
GRK2: G protein-coupled receptor kinase 2
GRα: glucocorticoid receptor α
GSDMD: gasdermin D
HIF-1α: hypoxia-inducible factor 1α
HO-1: heme oxygenase 1
IGF2BP3: insulin-like growth factor 2 mRNA-binding protein 3
IgG: immunoglobulin G
IKK: IκB kinase
IL: interleukin
iNOS: inducible nitric oxide synthase
IκB: NF-κB inhibitory protein
JAK: Janus kinase
JNK: c-Jun N-terminal kinase
JUN: JUN oncogenic transcription factor
LC3: human microtubule-associated protein light chain 3
LIFR: leukemia inhibitory factor receptor
LLDT-8: (5R)-5-Hydroxytriptolide
LPS: lipopolysaccharide
MAPK: mitogen-activated protein kinase
MCL1: myeloid cell leukemia-1
MCP-1: monocyte chemoattractant protein 1
MD2: myeloid differentiation factor 2
MDA: malondialdehyde
MDP: monomer derivative
MIF: macrophage migration inhibitory factor
MMP: matrix metalloproteinase
mPGES-1: microsomal prostaglandin E synthase 1
mTOR: mammalian target of rapamycin
MyD88: myeloid differentiation factor 88
NFATc1: nuclear factor of activated T cells c1
NF‐κB: nuclear factor κ
NLRP3: nucleotide-binding domains and leucine-rich repeat pyrin domains containing 3
NO: nitric oxide
Nrf2: nuclear factor erythroid 2-related factor 2
NSAIDs: non-steroidal anti-inflammatory drugs
PBMC: peripheral blood mononuclear cells
PDK-1: phosphatidylinositol-dependent kinase 1
PGE-2: prostaglandin E2
PI3K: phosphatidylinositol-4,5-bisphosphate 3-kinase
PKA: protein kinase A
PLC‐γ1: phospholipase C γ1
PYK2: proline-rich tyrosine kinase 2
RA: rheumatoid arthritis
RANK: receptor activator of NF-κB
RANKL: receptor activator of NF-κB ligand
ROS: reactive oxygen species
RSK2: ribosomal S6 kinase 2
SIN: sinomenine
SOD: superoxide dismutase
STAT: Signal transducers and activators of transcription
TGF-β: transforming growth factor-β
TGP: total glucosides of paeony
Th17: T helper cell 17
TLR4: toll-like receptor 4
TNF-α: tumor necrosis factor α
TP: triptolide
TRAF6: TNF receptor-associated factor-6
Treg: T regulatory cells
TREM: trigger receptors expressed on myeloid cell
VEGF: vascular endothelial growth factor
